# A New Chinese Medicine Intestine Formula Greatly Improves the Effect of Aminosalicylate on Ulcerative Colitis

**DOI:** 10.1155/2017/7323129

**Published:** 2017-11-20

**Authors:** Baohai Liu, Xuehua Piao, Lianyi Guo, Guijun Wang, Weihua Sun, Leming Gao, Xuefeng Zheng, Yanli Fang

**Affiliations:** ^1^Department of Gastroenterology, The First Affiliated Hospital of Jinzhou Medical University, Jinzhou 121001, China; ^2^Department of Traditional Chinese Medicine, The First Affiliated Hospital of Jinzhou Medical University, Jinzhou 121001, China; ^3^Administrative Logistics, The 2nd Dental Center, Peking University School of Stomatology, Beijing 100101, China

## Abstract

Ulcerative colitis (UC) is a chronic lifelong inflammatory disorder of the colon. Current medical treatment of UC relies predominantly on the use of traditional drugs, including aminosalicylates, corticosteroids, and immunosuppressants, which failed to effectively control this disease's progression and produced various side effects. Here, we report a new Chinese medicine intestine formula (CIF) which greatly improved the effect of mesalazine, an aminosalicylate, on UC. In the present study, 60 patients with chronic UC were treated with oral mesalazine alone or in combination with CIF enema. The combination of mesalazine and CIF greatly and significantly improved the clinical symptoms and colon mucosal condition and improved the Mayo Clinic Disease Activity Index and health-related quality of life, when compared to mesalazine alone. In particular, the addition of CIF further decreased serum levels of tumor necrosis factor-alpha and hypersensitivity C-reactive protein but in contrast increased interleukin-4. Thus, the results demonstrate the beneficial role of CIF in UC treatment, which may be mediated by the regulation of inflammation.

## 1. Introduction

Ulcerative colitis (UC) is a chronic and relapsing inflammatory disease caused by inflammation and sores in the lining of the large intestine and characterized clinically by recurrent episodes of bloody diarrhea, cramping, and abdominal pain and histologically by mucosal inflammation and injury [1, 2]. UC was reported to affect 120 to 200 per 100,000 people throughout the western world [3]. Recent reports also showed that the prevalence of UC in Asia was growing rapidly, including China [4, 5], Japan [6], and South Korea [7]. Although the pathogenesis mechanisms of UC are not completely understood, it is suggested that dysregulation of the pro/anti-inflammatory systems and antioxidant systems may be an important cause [2, 8].

Currently, the medical treatment of UC relies mainly on traditional drugs: aminosalicylates, corticosteroids, and immunosuppressants. These drugs reduce inflammatory injury and attenuate the expression of some proinflammatory molecules, but their side effects often result in reduced health-related quality of life and poor life satisfaction, particularly during long-term treatment [9, 10]. Therefore, there is an increasing interest in identifying alternative and more tolerable treatments for this disease.

Many traditional Chinese medicinal formulas have been proved to have beneficial effects on UC [11–15]. Here, we reported a new Chinese medicine intestine formula (CIF) for the treatment of UC. This CIF contains seven Chinese medicinal herbs: Radix Astragali Mongolici, Indigowood Root, Indigowood Leaf, Endoconcha Sepiae,* Bletilla striata*,* Cirsium japonicum*, and Common Cephalanoplos Herb. In the present study, the CIF greatly enhanced the effect of the traditional aminosalicylate mesalazine on UC patients including intestinal and extraintestinal symptoms.

## 2. Materials and Methods

### 2.1. Subjects

A total of 60 patients with left-sided UC were recruited from the Gastroenterology Department of the First Affiliated Hospital of Jinzhou Medical University from August 2011 through July 2016. These patients had at least 6-month mild-to-moderate UC history based on Truelove and Witts criteria. The exclusion criteria include the following: (1) pregnant or breastfeeding patients; (2) patients with a history of alcohol or drug abuse; (3) recent malignancy, significant medical illness, or concurrent medication; (4) patients on an antidepressant drug; (5) patients with inability to complete the questionnaires and those with a psychiatric illness. These patients received treatment with mesalazine (control group) or mesalazine plus CIF enema (CIF group). Details of age, sex, weight, heart rate, body temperature, and others are shown in Table 1. The study protocol was approved by the Ethics Committee of the First Affiliated Hospital of Jinzhou Medical University (Jinzhou, China). All participants provided written informed consent for this study.

### 2.2. Preparation of CIF

The CIF is composed of seven Chinese medicinal herbs: Radix Astragali Mongolici (50 g), Indigowood Leaf (50 g), Indigowood Root (50 g), Endoconcha Sepiae (30 g),* Bletilla striata* (30 g),* Cirsium japonicum* (10 g), and Common Cephalanoplos Herb (10 g). These herbs were immerged in 1000 ml of cool water and filtered. The filtrates were concentrated into a solution containing 1 g/ml of crude drugs and kept at 4°C until use. Patients in both control and CIF groups received oral mesalazine 1 g, 4 times daily for 8 weeks. Patients in CIF group were additionally treated with CIF enema (100 ml at bedtime) once daily during the 8 weeks of mesalazine treatment.

### 2.3. Colonoscopy Scores

Colonoscopy examination was performed before and after 8-week treatment. An Endoscopy Index was calculated by an experienced endoscopist. According to Mayo Endoscopic Score [16], the Endoscopic Index score (0–3 points) includes four categories: normal or inactive disease (0 points); erythema, decreased vascular pattern, and mild friability (1 point); marked erythema, absent vascular pattern, friability, and erosions (2 points); spontaneous bleeding and ulceration (3 points).

### 2.4. Score of Mayo Clinic Disease Activity Index

All patients received a medical evaluation with the Mayo Clinic Score System [17] before treatment and after 8-week treatment. Mayo Clinic Score System is one of the most commonly used activity indices for UC evaluation, in which Mayo Clinic Disease Activity Index (MCDAI) is counted based on the scores from four parameters (0–3 points each): stool frequency, rectal bleeding, endoscopic findings, and physician's global assessment. Thus, it can comprehensively evaluate the situation of UC patients. The MCDAI scores range from 0 to 12 points. Lower score refers to better health condition. According to the Mayo Clinic Score System, a clinical remission is defined as MCDAI 0–2 points with no individual subscore >1; an endoscopic remission is defined as endoscopic findings scored 0 or 1; and a clinical exacerbation is defined as 5 points together with an increase of endoscopic score of at least 1 point [14].

### 2.5. Assessment of Health-Related Quality of Life (HRQoL)

We used the inflammatory bowel disease questionnaire (IBDQ) to assess HRQoL [18]. This disease-specific questionnaire comprises 32 questions which are divided into four health subscales: bowel symptoms (10 questions); systemic symptoms, including sleep disorders and fatigue (5 questions); emotional functions such as depression, aggression, and irritation (12 questions); and social function, meaning the ability to participate in social activities and to work (5 questions). The participants choose one from seven graded responses in each question (score: 1–7 points). Consequently, the total scores range from 32 to 224 points. Lower scores indicate worse HRQoL.

### 2.6. Scores of Clinical Symptoms

Clinical symptoms associated with UC were evaluated before and after 8-week treatment. The clinical symptoms include diarrhea, bloody stool, mucous stool, abdominal pain, abdominal distention, and tenesmus. Symptoms were scored by the following specific criteria: 0, no clinical symptoms; 3, minor symptoms with small effects on HRQoL; 6, moderate clinical symptoms with significant impairment in daily function; 9, severe clinical symptoms and severe debilitation of patients in terms of daily function.

### 2.7. Cure Standards

*Cured*. Clinical symptoms vanished and the mucosa shows normal tissue by colonoscopy.* Significant Improvement*. Clinical symptoms vanished and the colonoscopy result shows that the mucosal lesions are significantly improved.* Effective*. Clinical symptoms vanished and the colonoscopy result shows mild inflammation of the mucosa or false mucosal polyp formation.* Ineffective*. There is no improvement in both clinical symptoms and colonoscopy.

### 2.8. Measurement of Serum Tumor Necrosis Factor-Alpha (TNF-*α*), Interleukin-4 (IL-4), and Hypersensitivity C-Reactive Protein (hs-CRP)

Serum levels of TNF-*α*, IL-4, and hs-CRP before and after 8-week treatment were measured with ELISA. Blood samples (5 ml each) were collected from all patients. Serum was acquired following centrifugation at 2,000*g* for 10 min at 4°C and aliquoted. The aliquots were stored at −20°C until use. The serum levels of TNF-*α*, IL-4, and CRP were measured using ELISA kits (cats. numbers EK0525, EK0404, and EK1316, resp.; Wuhan Boster Biological Engineering Co., Ltd., Wuhan, China), according to the manufacturer's protocols.

### 2.9. Erythrocyte Sedimentation Rate (ESR) Measurement

ESR was measured with an automated ESR analyzer.

### 2.10. Statistical Analysis

Data are presented as means ± SEM. Statistical analysis was performed with SPSS 13.0 software (SPSS Inc., Chicago, IL, USA). One-way ANOVA and Tukey's test were used for group comparisons. *P* < 0.05 was considered statistically different.

## 3. Results

### 3.1. CIF Improves Clinical Symptoms

**Figure 1:**
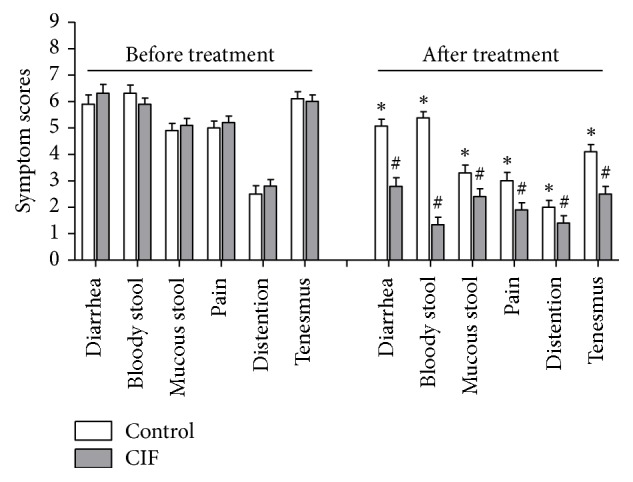
Effect of CIF on clinical symptoms of UC. Clinical symptoms associated with UC were evaluated in UC patients before and after 8-week mesalazine alone (control group) treatment or in combination with CIF enema (CIF group). Please note that CIF further and significantly reduced the symptom scores of all six symptoms (*n* = 30). ^*∗*^*P* < 0.05 versus before treatment; ^#^*P* < 0.05 versus control.

The main clinical symptoms of UC include diarrhea, bloody stool, mucous stool, abdominal pain, abdominal distention, and tenesmus. Thus, we first evaluated whether CIF improved these symptoms when added to mesalazine treatment (Figure 1). The scores of each symptom before treatment were not significantly different between mesalazine alone (control group) and mesalazine plus CIF enema (CIF group). After 8-week treatment, mesalazine alone moderately decreased the scores of all six symptoms (*P* < 0.05 versus before treatment). The addition of CIF significantly and markedly decreased the scores of all symptoms, when compared to the control group (*P* < 0.05). Thus, these results reveal an effective therapy of CIF on UC when in combination with mesalazine.

### 3.2. CIF Improves Mucosal Healing

**Figure 2:**
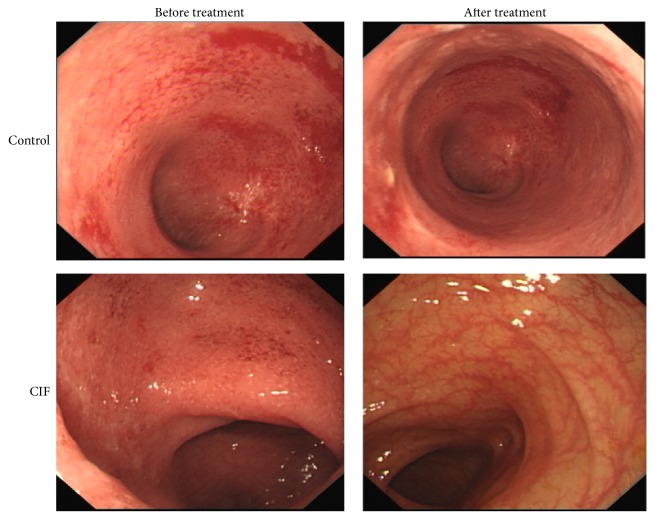
CIF improves mucosal healing. A representative endoscopic picture showing the mucosal condition before and after 8-week mesalazine treatment alone (control group) or in combination with CIF enema (CIF group).

We next examined whether the clinical effect of CIF was a consequence of mucosal healing. In the endoscopic examination, we observed a marked improvement of mucosal condition in both groups after 8-week treatment. As shown in Figure 2, mesalazine alone alleviated the mucous hyperemia. Mesalazine plus CIF caused a more marked improvement of mucous hyperemia, compared to mesalazine alone. The surface of the colon became smooth, and angiogenesis appeared in the impaired mucous. When endoscopic results from the 60 patients were scored, the data in all score grades (0–3) was not markedly different before treatment (Table 2). However, after 8-week treatment, there was a trend that more patients achieved mucosal healing in CIF plus mesalazine group than in mesalazine alone group. In CIF plus mesalazine group, 43.33% of the patients recovered completely (0 point), compared to 20% in mesalazine alone group. In score 1, the percentage was also bigger in CIF plus mesalazine group than in mesalazine alone group. In higher scores (2 and 3), which indicate worse mucosal conditions, the percentages were reversed. Thus, the results show that CIF greatly improves the mucosal condition of UC patients when added to mesalazine treatment.

### 3.3. CIF Improves Mayo Clinic Disease Activity Index

**Figure 3:**
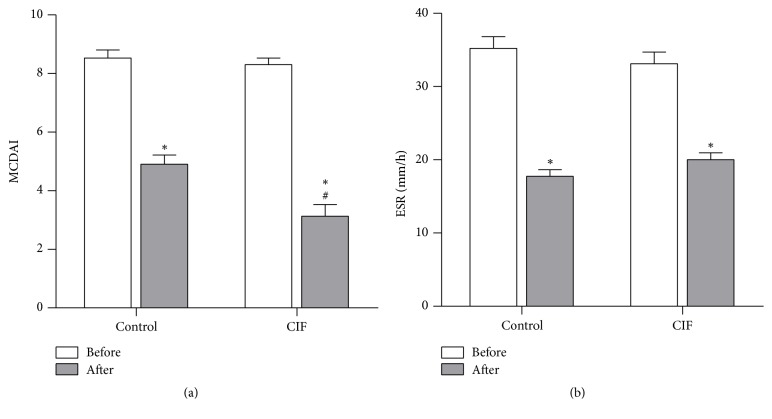
Effect of CIF on MCDAI and ESR. MCDAI associated with UC (a) and ESR (b) were evaluated in UC patients before and after 8-week mesalazine treatment alone (control group) or in combination with CIF enema (CIF group) (*n* = 30). ^*∗*^*P* < 0.05 versus before treatment; ^#^*P* < 0.05 versus control.

We evaluated the therapy effect of CIF on UC using Mayo Clinic Score System. Similar to the results in clinical symptoms and mucosal healing, there was no significant difference of MCDAI values before treatment between mesalazine alone and CIF plus mesalazine group (Figure 3(a)). Mesalazine alone moderately decreased the MCDAI values (*P* < 0.05). The addition of CIF significantly and markedly increased the effect of mesalazine in MCDAI scores. Thus, these results further support the notion that CIF is beneficial for mesalazine treatment in UC patients.

The ESR was also similar between the two groups before treatment. The two treatments decreased the ESR at an almost similar degree (Figure 3(b)), indicating that both treatments improved the disease condition.

### 3.4. CIF Improves HRQoL

We then evaluated the effect of CIF on HRQoL. Four parameters, that is, bowel symptoms, systemic symptoms, emotional function, and social function, were scored (Table 3). The four scores and total score were not significantly different before treatment between the two groups. Mesalazine alone moderately increased all scores including the total score. CIF further and significantly increased these scores (*P* < 0.05 versus control). Thus, the results, especially the improvements of extraintestinal characterizations, further support the beneficial effect of CIF on UC.

### 3.5. CIF Increases Total Efficacy

The effective rates were used to further evaluate the therapy effect of CIF after 8-week treatment. As shown in Table 4, the cure rate and significantly effective rate were markedly higher in CIF plus mesalazine group, compared to mesalazine alone group. Inversely, the ineffective rate was lower in CIF plus mesalazine group than in mesalazine alone group. The total effective rate was 93.3% and 73.3% in CIF plus mesalazine group and mesalazine alone group, respectively, and revealed a significant difference (*P* < 0.05). These results demonstrate that CIF increases the therapy effect of mesalazine on UC treatment.

### 3.6. Effect of CIF on Cytokines Levels and hs-CRP

**Figure 4:**
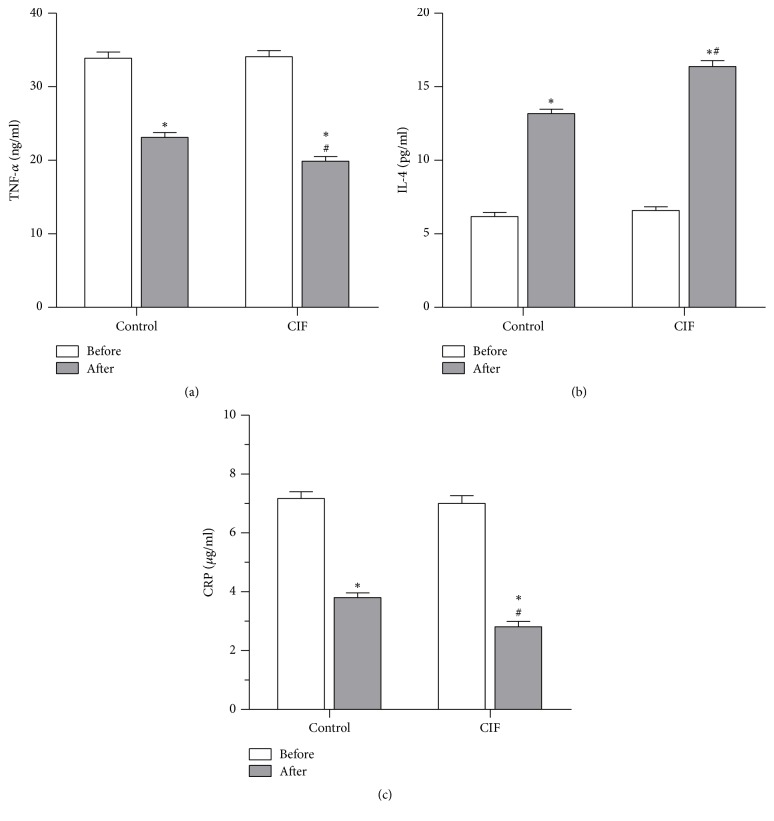
Effect of CIF on serum CRP and cytokines. Sera were obtained from patients before and after 8-week mesalazine treatment alone (control group) or in combination with CIF enema (CIF group) (*n* = 30). The levels of TNF-*α* (a), IL-4 (b), and hs-CRP (c) were measured with ELISA. ^*∗*^*P* < 0.05 versus before treatment; ^#^*P* < 0.05 versus control.

These results above demonstrate the beneficial role of CIF in the treatment of UC. Next, we explored the possible mechanisms underlying it. UC is a type of inflammatory bowel disease. Proinflammatory cell infiltration and proinflammatory cytokines (like TNF-*α* and IL-1*β*) release are considered as important events in UC [2, 19]. In particular, some anti-TNF-*α* reagents showed a potential effect on UC treatment [20]. Thus, we examined whether CIF alleviates TNF-*α* release. As shown in Figure 4(a), mesalazine alone decreased serum level of TNF-*α*. CIF plus mesalazine further and significantly decreased the serum level of TNF-*α*, compared to mesalazine alone (*P* < 0.05). IL-4, an anti-inflammatory cytokine, was also reported to play an important role in the development of UC [21, 22]. Mesalazine treatment increased the serum level of IL-4. The addition of CIF further and significantly increased the serum level of IL-4, compared to mesalazine alone (Figure 4(b), *P* < 0.05). We next examined whether CIF may change another UC-related factor, hs-CRP level in serum. Like TNF-*α* results, mesalazine alone revealed a marked inhibitory effect on the serum level of hs-CRP, and CIF plus mesalazine further and significantly decreased its level compared to mesalazine alone (Figure 4(c), *P* < 0.05).

## 4. Discussion

The present study was set forth to evaluate the therapy effect of a new Chinese herb formula CIF on UC when in combination with mesalazine and the underlying mechanisms. In our results, although mesalazine alone exhibited a therapy effect on UC patients, the addition of CIF further and significantly improved intestinal symptoms, mucosal condition, and extraintestinal characterizations. In particular, CIF decreased the serum level of proinflammatory factor TNF-*α* and increased the serum level of anti-inflammatory factor IL-4. These results demonstrated a therapy effect of CIF on UC, and anti-inflammatory activity may underlie the action mechanisms.

Chinese herbal medicine is widely used in the treatment of UC. In an analysis of 10,218 UC cases in China, 20.1% of the patients were treated with Chinese herbs, and 59.1% were treated with combined Chinese and western medicines (like mesalazine and/or corticosteroids) [23]. Thus, Chinese herbal medicine may offer an exciting potential for discovering new agents for UC treatment. Indeed, some pure and crude extractions from Chinese medicine herbs have been proved to have a therapy effect on UC in some experimental animal models [2, 15]. In the present study, we combined a Chinese herbal medicine formula CIF with classical mesalazine to treat UC patients. The 8-week treatment significantly improved the intestinal symptoms, mucosal condition, and extraintestinal characterizations in UC patients, compared to mesalazine alone, showing good therapy effect on UC. Among the seven components of this CIF, Radix Astragali Mongolici was used in another famous Chinese medicine formula to treat various gastrointestinal tract diseases, such as gastritis and stomach ulcer [24]. Indigowood Root has been used in traditional medicine for its potential anti-inflammatory effect. Indigowood Root protects against radiation-caused damage in the hematopoietic system with a significant reduction of serum TNF-*α*, IL-1beta, and IL-6 [25]. Endoconcha Sepiae is a classical herbal medicine used in gastrointestinal diseases. It was reported to have a gastroprotective potential against indomethacin [26].* Bletilla striata* has been not only widely used for the treatment of hematemesis, hemoptysis, and traumatic bleeding due to the efficacy of arresting bleeding with astringent action, but also topically applied to overcome ulcers, sores, swellings, and chapped skin due to the efficacy of dispersing swelling and promoting tissue regeneration [27]. An extract from* Bletilla striata* has been proved to have a good effect on wound healing [28].* Cirsium japonicum* has been employed traditionally in the treatment of inflammatory symptoms. Its extracts and principal ingredient apigenin were reported to have a strong antioxidant activity [29, 30]. Luteolin, another major component of* Cirsium japonicum*, reduced D-galactosamine/lipopolysaccharide-stimulated high serum level of TNF-*α* and protein expression of TNF-*α* receptor-associated death domain [31]. These pharmacological effects of major components of CIF collectedly may contribute to the therapy results in UC patients in the present study.

Consistent with the anti-TNF-*α* effect of luteolin, one major component of* Cirsium japonicum* in D-galactosamine/lipopolysaccharide-induced liver damage model [31], this CIF also significantly reduced serum level of TNF-*α* in UC patients. As proinflammatory cell infiltration and proinflammatory cytokine (like TNF-*α*) release are considered as important events in UC [2, 19], thus, the inhibition of TNF-*α* by CIF in the present study can be considered as an important action mechanism in UC treatment. Especially interesting is that our results showed that CIF in combination with mesalazine further increased the serum level of IL-4, compared to mesalazine alone. IL-4, as an anti-inflammatory factor, was increased in UC when effective treatments were given in some UC experimental models [32, 33]. Therefore, the increased anti-inflammatory factor IL-4 by CIF may become another important mechanism of CIF in UC treatment.

ESR and hs-CRP were used as a measure of the acute phase response in UC [34]. In particular, hs-CRP increased significantly when exacerbation of colitic symptoms occurred [35]. And effective treatment of Crohn's disease and UC significantly decreased the serum level of hs-CRP [36]. Consistent with the previous reports, our results showed that CIF further decreased the serum level of hs-CRP compared to mesalazine alone, indicating a good treatment effect of CIF on UC.

In conclusion, the present results demonstrate that CIF exerted a beneficial effect on UC patients in combination with classical mesalazine. The suppression of TNF-*α* and hs-CRP and increase of IL-4 may underlie the action mechanism of CIF.

## Figures and Tables

**Table 1:** General patient information before mesalazine treatment alone (control group) or in combination with CIF enema (CIF group).

Parameters	Control	CIF	*P* value
Patients (males/females)	13/12	16/9	
Age (years)	45.07 ± 14.44	47.11 ± 12.08	0.576
Weight (kg)	60.88 ± 9.05	60.97 ± 9.99	0.974
Height (cm)	168.82 ± 5.49	163.62 ± 7.70	0.221
Disease duration (yr)	5.48 ± 2.12	5.16 ± 3.25	0.712
Heart rate/min	76.07 ± 4.81	76.42 ± 6.39	0.816
Systolic BP (mmHg)	117.00 ± 13.23	120.84 ± 14.97	0.285
Diastolic BP (mmHg)	72.53 ± 10.35	73.07 ± 11.65	0.846
Body temperature (°C)	36.48 ± 0.19	36.54 ± 0.22	0.313
Serum CRP (*μ*g/mL)	5.86 ± 0.82	6.13 ± 1.27	0.261

**Table 2:** Mucosal scores before and after 8-week mesalazine treatment alone (control group) or in combination with CIF enema (CIF group).

	Mucosal score	Control (*n*, %)	CIF (*n*, %)
Before treatment	0	0/0%	0/0%
	1	7/23.3%	6/20%
	2	20/66.7%	18/60%
	3	3/10%	6/20%

After treatment	0	6/20.00%	13/43.33%
	1	8/26.67%	12/40%
	2	11/36.67%	5/16.67%
	3	5/16.67%	0/0.00%

**Table 3:** HRQoL scores before and after 8-week mesalazine treatment alone (control group) or in combination with CIF enema (CIF group).

	Before treatment		After treatment	
	Control	CIF	Control	CIF
Bowel symptoms	40.43 ± 3.34	40.77 ± 3.08	42.7 ± 4.25	46.97 ± 6.68
Systemic symptoms	19.2 ± 3.03	20.23 ± 2.12	21.67 ± 3.13	24.07 ± 5.32
Emotional function	48.7 ± 8.63	48.7 ± 9.08	57 ± 8.72	61.5 ± 10.06
Social function	20.57 ± 4.01	21.03 ± 3.37	22.30 ± 2.93	26.13 ± 6.23
Total	128.9 ± 11.86	130.73 ± 12.37	143.67 ± 11.23	158.67 ± 19.08

**Table 4:** Effective rate after 8-week mesalazine treatment alone (control group) or in combination with CIF enema (CIF group).

	Control (*n*/%)	CIF (*n*/%)
Cured	3/10%	10/33.3%
Significantly effective	6/20%	12/40%
Effective	13/43.3%	6/20%
Ineffective	8/26.7%	2/6.7%
Total effective rate	30/73.3%	30/93.3%
